# Climate informed precision health

**DOI:** 10.3389/fpubh.2026.1805939

**Published:** 2026-04-28

**Authors:** Yvette P. Conley, Nada Lukkahatai, Marilyn J. Hammer, Rose Mary Xavier, Susan W. Wesmiller, Hudson P. Santos, Ruth Lucas, Angela Starkweather

**Affiliations:** 1 School of Nursing, University of Pittsburgh, Pittsburgh, PA, United States; 2 Johns Hopkins School of Nursing, Baltimore, MD, United States; 3 Department of Nursing and Patient Care Services and Department of Medical Oncology, Division of Population Sciences, Dana-Farber Cancer Institute, Boston, MA, United States; 4 School of Nursing, Northeastern University Bouvé College of Health Sciences, Boston, MA, United States; 5 Cizik School of Nursing, McGovern Medical School, and School of Behavioral Health Sciences, The University of Texas Health Science Center at Houston, Houston, TX, United States; 6 University of Miami School of Nursing and Health Studies, Coral Gables, FL, United States; 7 University of Connecticut Elizabeth Deluca School of Nursing, Storrs, CT, United States; 8 School of Nursing, Rutgers University, Newark, NJ, United States

**Keywords:** climate, climate-informed, omics, personalized health care, precision health care

## Abstract

Precision healthcare requires incorporation of climate-related factors relevant to the patient. There is a critical need for healthcare systems to incorporate environment and community vital signs into electronic health records to facilitate incorporation of climate-related exposures into personalized patient care plans. Educators need to incorporate precision healthcare that includes climate-related assessments for patients into the education of current and future health care providers. Research focusing on precision healthcare that integrates data sources from omics and across the exposome, including climate-related data is needed. Practice guidelines need to be developed that incorporate the exposome, including climate-related health threats, into precision health at the healthcare system level and into personalized recommendations for patients.

## Introduction

**Figure 1:**
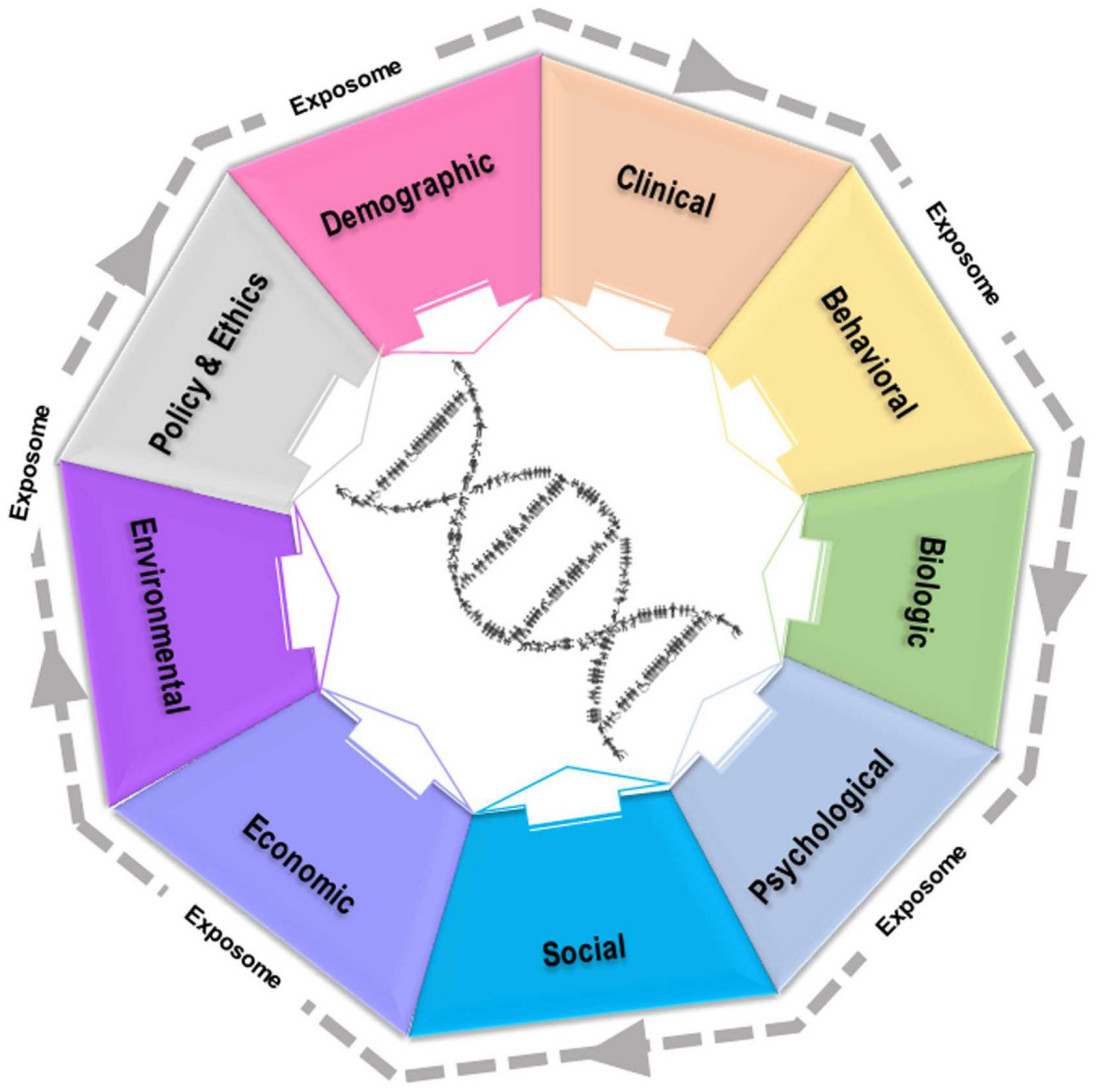
Precision health model emphasizing the exposome that includes climate informed precision health (1).

Precision healthcare realizes that one-size-fits-all approaches based on an “average” individual does not work well for most individuals and can even be harmful, especially if the “average” individual does not adequately represent the diversity of the population. Precision health has already revolutionized patient care, for example in the areas of oncology and pharmacology, primarily through taking omics variation into account. While omics variation is central, precision health goes beyond omics variation by taking the exposome (e.g., behavior, social, and environmental factors) into account which is exemplified in a recent precision health model (Figure 1) (1).

Climate-related factors are one environmental aspect of the exposome that is often overlooked in precision health care. Incorporating climate-related exposures into precision healthcare requires linking environmental data (e.g., heat exposure, air quality, cumulative toxin exposure assessment) with individual-level biological and clinical information. This integration allows clinicians to assess climate-related risk within the context of a patient's genetic susceptibility, health status, and social environment, enabling more personalized prevention and management strategies. In practice, this approach involves integrating environmental monitoring data with clinical and patient-level information within healthcare systems so that climate-related exposures can inform risk assessment, clinical-decision-making, and personalized care planning.

Two climate responsive healthcare frameworks that incorporate environmental exposures and health are the Precision Environmental Health (PEH) framework that evaluates the impact of interaction effects between genetics and exposures on health, and the Precision Ecologic Medicine (PCM) framework that is more expansive and incorporates community-based data into healthcare delivery. The PEH establishes the science of how genes and environment interact, while the PCM applies these findings in real-world healthcare settings. These frameworks provide a pathway for integrating environmental exposure data such as heat exposure or air quality into clinical risk assessment and personalized care planning within prevision healthcare.

This policy brief expands upon the PEH and PCM frameworks and advocates for policies and protocols that foster the inclusion of climate-related factors into precision health plans for patients as well as public health policies. The goal is to support education, research, practice, and policy that integrates climate considerations into precision healthcare and allow clinicians to identify and mitigate climate-related health threats. Additionally, internet-based resources linking climate to health for health care providers and policy makers can be found in Table 1.

**Table 1:** Internet-based resources linking climate to health for health care providers and policy makers.

Organization	Focus	Website
World Health Organization	Communicating on climate change and health: toolkit for health professionals	https://www.who.int/publications/i/item/9789240090224
The Medical Society Consortium on Climate and Health	Climate resilience and health	https://medsocietiesforclimatehealth.org/
Havard School of Public Health	Center for climate, health, and the global environment	https://hsph.harvard.edu/research/climate-health-c-change/outreach/building-climate-resilient-healthcare-clinics/
U.S. Department of Health & Human Services: Administration for Strategic Preparedness and Response	Technical Resources, Assistance Center, and Information Exchange (TRACIE)	https://asprtracie.hhs.gov/technical-resources/158/climate-change-and-healthcare-system-considerations/0
U.S. Department of Health & Human Services: Centers for Disease Control (CDC)	Climate and health	https://www.cdc.gov/climate-health/
U.S. Department of Health & Human Services: Centers for Disease Control (CDC)	Guide for clinicians on heat and health	https://www.cdc.gov/heat-health/hcp/clinical-guidance
CDC Foundation	Extreme weather and health resources	https://www.cdcfoundation.org/ExtremeWeather-Health/resources
Canadian Association of Nurses for the Environment	Nursing toolkit for planetary health	https://www.nursingtoolkit4planetaryhealth.ca/
Alliance of Nurses for Healthy Environments	Climate and health toolkit	https://climateandhealthtoolkit.org/
World Health Organization	Compendium of WHO and other UN guidance on health and environment	https://www.who.int/tools/compendium-on-health-and-environment
U.S. Department of Health & Human Services: National Institute of Environmental Health Sciences	Personalized Environment and Genes Study (PEGS)	https://www.niehs.nih.gov/research/atniehs/labs/crb/studies/pegs
U.S. Department of Health & Human Services: National Institute of Environmental Health Sciences	Human Health Exposure Analysis Resource (HHEAR)	https://www.niehs.nih.gov/research/supported/exposure/hhear
Lancet Countdown on Health and Climate Change	Monitoring and reporting on the health impacts of climate change	https://www.lancetcountdown.org/
National Oceanic and Atmospheric Administration (NOAA)	Climate data, heat and weather forecasting, and early warning systems relevant to climate-related health risk assessment and preparedness	https://www.noaa.gov/climate
World Meteorological Organization	Climate information and services in supporting health system preparedness, resilience, and adaptation	https://wmo.int/topics/health

## Evidence and research findings

### Climate related health threats

There are many climate-related threats to human health, including heat exposure, air quality, vector-borne infectious diseases, floods, and droughts. The frequency, intensity, and duration of extreme health events are increasing globally due to climate change factors and amplifying cumulative health risks across populations (2, 3). This policy brief uses two of these threats as exemplars to highlight the impact of climate on human health, focusing on heat exposure, and poor air quality. High heat exposure is associated with increased risk of stillbirth, premature birth, congenital anomalies, decreased exclusive breastfeeding (4), and other obstetric complications such as gestational diabetes mellitus (5, 6). In addition, significant links have been identified between high heat exposure and increased suicide incidence, mental health, and wellbeing (7, 8) anxiety (9), increased mental illness related hospitalizations (7) including for dementia, schizophrenia, and psychoses (10), allergic, airway, and autoimmune diseases (11, 12), and high heat exposure. Similarly, poor air quality is associated with alterations in leukocyte distribution (13) and brain volume (14); impaired mental health (8, 15–17) and increased risk for mental health disorders (18); and allergic and airway diseases (12). There is substantial crossover between high heat and poor air quality exposure with exposure, both linked to mental health (19), reproductive health (20), and cardiovascular health (21) related issues.

As shown in Figure 1, there are numerous interaction effects between multiple exposome domains and underlying omics profiles that influence health and disease across the lifespan (1). Specific to environmental exposures, a number of chemical toxins (e.g., 2,3,7,8-tetrachlorodibenzo-*p*-dioxin) contribute to transgenerational genetic alterations and associated health risks (2). These exposures to both men and women affect developing fetuses. While this may not directly affect the newborn, the epigenetic exposures throughout life, layered on top of inherent DNA alterations, increase the risk for adult-onset chronic diseases (2).

### Vulnerable populations to climate related health threats

While some individuals are more vulnerable to the impact of climate factors on their health, it is important to recognize that everyone's health is at risk from climate-related exposures and this risk fluctuates over the lifespan. Individuals most vulnerable to climate-related exposures are infants and young children (3, 4), older adults (5), individuals with chronic health conditions [e.g., heart disease; respiratory conditions such as asthma and chronic obstructive pulmonary disease (COPD); diabetes; mental illness], and exposures during pregnancy that impact maternal and fetal health (6–10). From a precision health perspective, these patterns of susceptibility reflect the convergence of biological vulnerability, cumulative environmental exposures, and social and structural determinants that shape individualized climate-related risk. Some individuals are at additional risk such as those who are socially isolated, live in under-sourced areas, and those relying on medications that increase risk to heat and air quality related illnesses. Failure to account for this heterogeneity in exposure and vulnerability risks obscuring high-risk subgroups and limiting the effectiveness of one-size-fits-all prevention and mitigation strategies.

While population-level public health policies aim to reduce environmental exposure broadly (e.g., improving air quality standards or reducing urban heat), these interventions primarily operate at community or policy level to lower overall environmental risk. In contrast, precision health focuses on clinical decision-making at the individual level, identifying patients who may be disproportionately affected by these exposures due to genetic susceptibility, medical conditions, medication use, or cumulative environmental exposures. These two approaches are complementary. Population-level interventions reduce baseline environmental risk, while precision healthcare allows clinicians to tailor prevention and management strategies for individuals at greater risk.

### Genetic-based vulnerability to climate related health threats

There are many genetic conditions that are exacerbated by climate-related health threats. One example is that high heat exposure is associated with variation in genes that cause malignant hyperthermia in response to anesthesia. We now know that these same variations place individuals at increased risk for heat-related illness and mortality. In fact, it is estimated that 1 in 300 to 1 in 3,000 individuals harbor a variant that increases their vulnerability to heat (11). As another example, high heat exposure from urban heat islands has been linked to increased risk for schizophrenia onset and to frontal and temporal lobe micro and macrostructural abnormalities particularly among individuals with higher polygenic risk (12).

Regarding air quality, a genetic variation in the gene that codes for a protein that protects lung tissue is associated with alpha-1 antitrypsin deficiency (AATD). One in 25 Americans of European ancestry are carriers of this variation increasing their risk of significantly reduced lung function at an early age. In addition, for these individuals who are exposed to poor air quality, their risk for early onset emphysema (13) and lung damage (14) is significantly increased. Emerging evidence from population-based cohorts suggests that air pollution in the context of high polygenic risk also increases the risk for various mental health disorders, including depression and anxiety (15).

Vulnerability to climate-related health threats due to genetic variation is an important area where precision healthcare that incorporates omics and climate can have a great impact.

### Climate impacts health through epigenomics

Biological age acceleration has been associated with climate-related health threats (16–19). Biological aging refers to molecular changes at the cellular level which can differ substantially from chronological age. The difference between biological and chronological age is a measure of accelerated biological aging. Accelerated biological aging is associated with increased risk for early onset of age-related conditions and shortening of healthspan and lifespan. Evaluating the epigenome using epigenetic clock algorithms provides one approach to measuring biological age and is termed epigenetic aging (20). One of the factors that promotes epigenomic age acceleration (EAA) is climate-related exposures across the lifespan, including while in utero (21). Two large studies [*n* = 3,947; US Health and Retirement Study (22) and *n* = 2,084; Taiwan biobank representing the general population (18)] demonstrate the relationship between high ambient temperature and EAA. Beyond accelerated aging, epigenomic changes in response to high heat exposure and poor air quality have been documented across developmental stages. Higher land surface temperatures are associated with bone growth in utero and into childhood and this relationship is mediated by epigenomic changes (23). Epigenomic changes in response to poor air quality have been well documented (6, 24–27) with some implicating increased risk for neurodevelopmental conditions such as autistic spectrum disorder (28), attention-deficit hyperactivity disorder (ADHD) (24), mental health disorders (29), cancer, neurodegenerative conditions such as Alzheimer's disease (28), metabolic diseases (26, 30), airway related disease (6, 31), and cardiovascular disease (4).

## Actionable recommendations and policy options

Evidence supports the impact of climate factors on human health and the need to incorporate climate information into precision health initiatives and personalized healthcare needs to be a goal. We have actionable recommendations for healthcare providers and policy makers to accomplish this goal.

- Incorporate environment (e.g., heat exposure, air quality, cumulative toxin exposure assessment) and community (e.g., access to transportation, walkability score) vital signs into electronic health records to facilitate incorporation of climate-related exposures into personalized patient care plans.
- Embed climate-related health risk assessment and mitigation of those risks into clinical guidelines and health care compensation structures.
- Integrate climate-related health assessments into the precision health education of current and future health care providers.
- Prioritize research focused on precision health care that integrates data sources from omics and across the exposome, including climate related data.
- Develop clinical decision support tools that translate climate and exposome data into actionable, individualized risk assessments. For example, automated alerts could notify providers when patients with chronic respiratory disease are exposed to hazardous air quality or when individuals taking medication that impair thermoregulation face elevated heat risk.
- Establish data standards and interoperability frameworks to support integration of climate, environmental, and clinical data across systems.
- Develop practice guidelines that incorporate the exposome, including climate-related health threats, into precision health at the healthcare system level and into personalized recommendations for a patient.

## Data Availability

The original contributions presented in the study are included in the article/supplementary material, further inquiries can be directed to the corresponding author.
